# LncRNA DQ786243 affects Treg related CREB and Foxp3 expression in Crohn’s disease

**DOI:** 10.1186/1423-0127-20-87

**Published:** 2013-12-01

**Authors:** Yu Qi Qiao, Mei Lan Huang, An Tao Xu, Di Zhao, Zhi Hua Ran, Jun Shen

**Affiliations:** 1 Division of Gastroenterology and Hepatology, Ren Ji Hospital, School of Medicine, Shanghai Jiao Tong University, Shanghai Institute of Digestive Disease, Shanghai 200127, China; 2 Shanghai Inflammatory Bowel Disease Research Center, Shanghai Jiao Tong University, Shanghai 200127, China; 3 Key Laboratory of Gastroenterology & Hepatology, Ministry of Health, Shanghai Jiao Tong University, Shanghai 200127, China

## Abstract

**Background:**

Long non-coding RNAs (lncRNAs) have different functions in cells. They work as signals, decoys, guides, and scaffolds. Altered lncRNA levels can affect the expression of gene products. There are seldom studies on the role of lncRNAs in inflammatory bowel disease (IBD).

**Results:**

Quantitative RT-PCR showed that DQ786243 was significantly overexpressed in clinical active CD patients compared with clinical inactive CD patients (*P* = 0.0118) or healthy controls (*P* = 0.002). CREB was also more highly expressed in active CD than in inactive CD (*P* = 0.0034) or controls (*P* = 0.0241). Foxp3 was interestingly lower in inactive CD than in active CD (*P* = 0.0317) or controls (*P* = 0.0103), but there were no apparent differences between active CD and controls. CRP was well correlated with DQ786243 (*r* = 0.489, *P* = 0.034), CREB (*r* = 0.500, *P* = 0.029) and Foxp3 (*r* = 0.546, *P* = 0.016). At 48 hours after DQ786243 transfection, qRT-PCR showed both CREB (*P* = 0.017) and Foxp3 (*P* = 0.046) had an increased mRNA expression in Jurkat cells. Western blot showed the same pattern. After DQ786243 transfection, CREB phosphorylation ratio (p-CREB/t-CREB) was increased (*P* = 0.0043).

**Conclusion:**

DQ786243 can be related with severity of CD. It can affect the expression of CREB and Foxp3 through which regulates the function of Treg. CREB itself seems not the mediator of DQ786243 to up-regulate Foxp3. The phosphorylation of CREB might play a more important role in the process.

## Background

Long non-coding RNAs (lncRNAs) are non-protein coding transcripts over 200 bases in length. In functional genomics, they belong to a new type of regulatory genes. Compared with micro-RNAs (mi-RNAs) [1], their characteristics are much less known. More and more evidences show that lncRNAs play critical roles in cells, including stem cell development, pluripotency, cell growth and apoptosis, etc. [2-6]. LncRNAs regulate protein coding gene at the level of chromatin remodeling, transcriptional control and post-transcriptional processing [7]. They work as signals, decoys, guides, and scaffolds [8]. They may “sponge” mi-RNAs to regulate the gene expression [9]. Many researches showed altered lncRNA levels could affect the expression of gene products. Implication of lncRNAs has been found in nervous system diseases [10,11]. LncRNAs also play critical roles in cancer biology [12,13]. However, there are seldom studies on the role of lncRNAs in inflammatory bowel disease (IBD).

LncRNA DQ786243 (mentioned as DQ786243 thereafter) is a new lncRNA, which has been found overexpressed in hepatocellular carcinoma (HCC) [14]. The function of DQ786243 is not fully understood. In our preliminary work, we analyzed a small amount of lncRNA expression microarrays in Crohn’s disease (CD) patients and healthy controls. Results accidentally showed that the expression of DQ786243 was closely related with the expression of cAMP response element binding protein (CREB), which is important for the activity of the TCR response element in the forkhead box P3 (Foxp3) [15], a master transcription factor in function and development of regulatory T cells (Treg) [16].

As a type of IBD, the etiology of CD has not been fully understood. An aberrant immune response to intestinal microflora might contribute to the disease [17]. Regulatory T lymphocytes (Tregs), defined as CD4 positive, Foxp3 expression T cells [18], are thought to keep balance in immunity and limit the inflammation. CD25 expression was once considered as a marker of Tregs until Foxp3 was found. Foxp3 was discovered as a “master control gene” for CD4+ Treg development and functional maturation [19,20]. FOX proteins are members of forkhead/winged-helix family which work as transcriptional regulators and may have similar DNA binding interactions in transcription process. Mutations of Foxp3 in human being can lead to severe condition, which is called IPEX (immune dysregulation, polyendocrinopathy, enteropathy, X-linked syndrome) [21]. Defects of Tregs could be an important pathogenesis of CD [22]. It might implicate the severity of the disease. In this study, we try to figure out the relationship between DQ786243 and Treg related genes expression in CD.

## Methods

### Patients and samples

19 CD patients and 9 healthy controls were enrolled in this study. Among these 19 patients, 11 of them were clinical active and 8 of them were clinical inactive (See Table 1). The diagnoses were confirmed based on clinical endoscopic, pathological and serological examinations by three gastroenterologists. Pregnant patients and patients with other chronic diseases or cancer were not included. The activity assessment of the disease was based on Harvey-Bradshaw Index (HBI) [23]. Total score < 5 stands for clinical inactive disease, ≥ 5 stands for clinical active disease. Montreal classification were also used in estimation of disease characteristics [24]. Healthy blood donors were healthy physicians and postgraduate students from Renji Hospital. Whole blood samples of donors were collected in EDTA anti-coagulated vacutainer tubes and Ficoll density gradient (Sigma Aldrich, St. Louis, MO, USA) was used in isolating peripheral blood mononuclear cells (PBMCs) from whole blood. Samples of PBMC were stored in RNA Later (Qiagen, Hilden, Germany) in -80°C followed the manufacturer’s instructions. All donors were well informed and the process were approved by Ethics Committee of Renji Hospital, School of Medicine, Shanghai Jiao Tong University (Shanghai, China).

**Table 1:** Characteristic of patients with inflammatory bowel disease and healthy controls

	**Active CD (n = 11)**	**Inactive CD (n = 8)**	**CTL (n = 9)**	**P value**
Age (yrs)	31.27 ± 8.98	31.13 ± 9.16	31.11 ± 8.82	0.999*
BMI (kg/m^2^)	21.55 ± 1.97	21.63 ± 2.62	23.56 ± 1.74	0.0872*
Gender (Female/Male)	4/7	3/5	4/5	
Smoking	1/11	0/8		
Extent**				
L1	1	3		
L2	1	1		
L3	9	4		
Lx+L4	1	0		
Behavior**				
B1	6	2		
B2	3	3		
B3	2	3		
Bx+P	5	0		

*One way ANOVA; **Montreal classification of extent and behavior of CD.

CD = Crohn’s Disease; CTL = control.

### Cell line and transfection

Jurkat cells (Boster, Wuhan, China) were grown in Roswell Park Memorial Institute-1640 medium (RPMI-1640) (Gibco, Carlsbad, CA, USA) with 10% fetal bovine serum (FBS) (Hyclone, Logan, UT, USA) and antibiotics at 37°C and 5% CO_2_. Transient transfection was performed and the manufacturer’s instructions were followed to use Lipofectamine reagent (Invitrogen, Carlsbad, CA, USA). Typically, 5 × 10^6^ cells/well in 6-well dishes were transfected with 0.5 μg of plasmids. Overexpressed DQ786243 transfection plasmid was purchased from SLNco (Shanghai, China). The plasmids were grown in bacteria following the standard techniques and purified with Qiagen Plasmid kit (Qiagen, Hilden, Germany). Control group was transfected with empty vector pLentiLox 3.7 (Fudan University, Shanghai, China) followed the same instruction of transient transfection. Cells were harvested 0 hour, 24 hours and 48 hours post transfection for further experiment.

### Quantitative real-time polymerase chain reaction (qRT-PCR)

Primers were designed with PRIMER 5.0 (ABI, Foster City, CA, USA) and synthesized by Generay (Generay, Shanghai, China). Total RNA from PBMCs or Jurkat cells was extracted with TRIzol (Invitrogen, Carlsbad, CA, USA), and cDNA was synthesized using PrimeScript RT reagent Kit (Takara Bio Inc, Shiga, Japan). The primers for DQ786243 were: forward, 5′-TAGGCGGACATTGTGGTGAGT-3′, and reverse 5′-CTTCTGCTGGGCTGTTGAGTG-3′; those for CREB were: forward, 5′-GGAGTGCCAAGGATTGAAGAAGA-3′, and reverse 5′-TGCTGTGCGAATCTGGTATGTT-3′; those for Foxp3 were: forward 5′-AGAAGCAGCGGACACTCAATG-3′, reverse 5’-GACTCAGGTTGTGGCGGATG-3′. Reverse transcription was performed at 37°C for 15 minutes and at 85°C for 5 seconds using the PrimeScript RT reagent kit (Takara Bio Inc, Shiga, Japan) following the manufacturer’s protocol. PCRs were performed 5 min at 95°C, followed by 45 cycles of 15 s at 95°C, and 1 min at 60°C. The processed were conducted on a Realtime Thermo Cycler (FTC2000, Funglyn, Canada) with SYBR Premix Ex Taq kit (TaKaRa Bio Inc., Shiga, Japan). The specificity of real-time PCR was confirmed by melting-curve analysis. Relative expressions were determined by normalizing expression of each target to GAPDH. Data were analyzed by 2-ΔΔCt method [25].

### Western blot

Cytoplasmic protein was extracted from Jurkat cells at 48 hours after transfection using nucleic/plasma protein extraction kit (Viagene, Tampa, FL, USA). Cytoplasmic protein is separated by SDS-polyacrylamide gel electrophoresis (SDS-PAGE) after quantitation. Samples are separated in SDS-PAGE for 1 hour respectively (Bio-rad, Hercules, CA, USA) and transferred onto a polyvinylidene fluoride membranes at 4°C. The membrane is blocked for 1 hour at room temperature in 5% non-fat dried milk, and then incubated with the primary antibody with continuous gentle agitation overnight at 4°C. Polyclonal rabbit anti-CREB and polyclonal rabbit anti-Foxp3 were purchased from Boster (Boster, Wuhan, China). Anti-phospho-CREB rabbit antibody p-CREB-1 (Ser-133) was purchased from Santa Cruz (Dallas, Texas, USA). Then the membranes are incubated for 1 hour with HRP-conjugated secondary anti-rabbit antibodies (Boster, Wuhan, China) at room temperature, and finally developed by SuperSignal West Pico Chemiluminescent Sub-strate (Pierce Biotechnology, Rockford, IL, USA).

### Statistics

GraphPad Prism 5.0 for Windows (GraphPad Software, San Diego CA, USA) was used in statistics. *P* < 0.05 was considered significant with ANOVA analysis, t-test and linear regression.

## Results

### Characteristics of included subjects

19 patients and 9 healthy controls were enrolled in our research. There were no significant age and BMI differences among three groups. One patient had a history of smoking. Montreal classification of extent and behavior of CD [24] were showed (Table 1).

### RNA levels of DQ786243, CREB and Foxp3 in PBMC from patients

**Figure 1:**
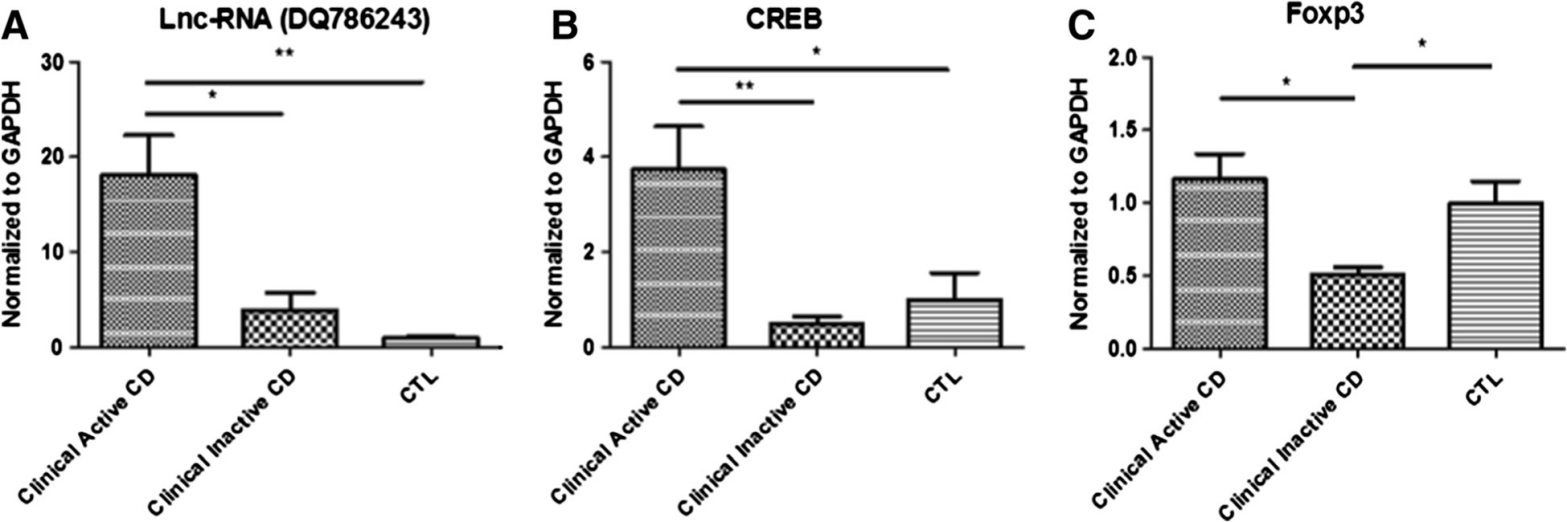
**DQ786243 was significantly overexpressed in clinical active CD patients compared with clinical inactive CD patients or healthy controls.** And there were no significant differences between inactive CD and controls **(A)**. CREB was also more highly expressed in active CD than in inactive CD or controls. No significant differences were found between inactive CD and controls **(B)**. Foxp3 was interestingly lower in inactive CD than in active CD or controls, but there were no significant differences between active CD and controls **(C)**. *P < 0.05, **P < 0.01.

Quantitative RT-PCR showed that DQ786243 was significantly overexpressed in clinical active CD patients (ANOVA, *P* = 0.004) compared with clinical inactive CD patients (t-test, *P* = 0.012) or healthy controls (t-test, *P* = 0.002). And there were no significant differences between inactive CD and controls (See Figure 1A). CREB was also more highly expressed in active CD (ANOVA, *P* = 0.005) than in inactive CD (t-test, *P* = 0.003) or controls (t-test, *P* = 0.024). No significant differences were found between inactive CD and controls (See Figure 1B). Foxp3 was interestingly lower in inactive CD (ANOVA, *P* = 0.027) compared to that in active CD (t-test, *P* = 0.032) or controls (t-test, *P* = 0.010), but there were no significant differences between active CD and controls (See Figure 1C).

### The relationship of RNA levels and clinical inflammation serological markers

**Figure 2:**
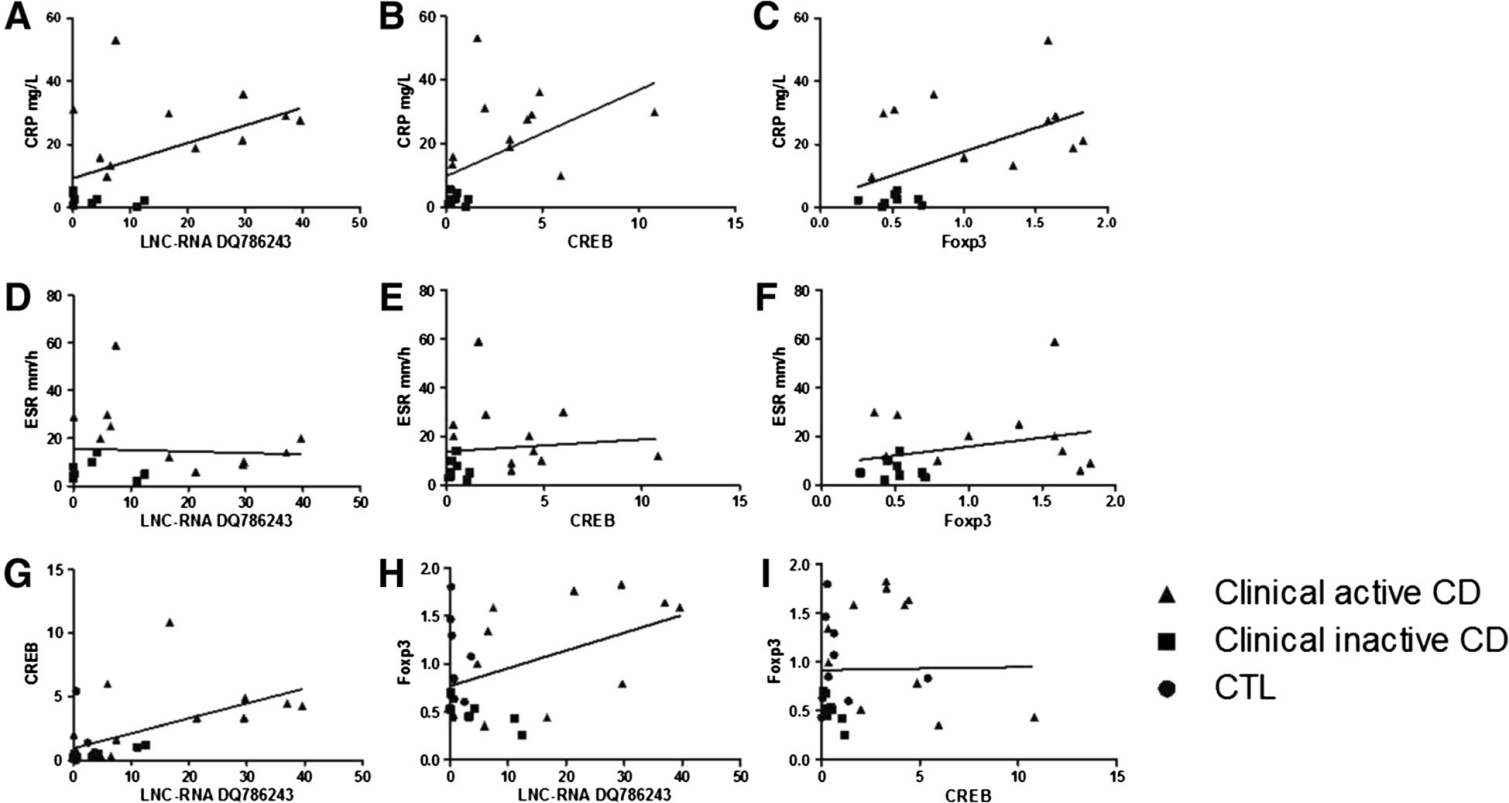
**The relationship of RNA levels and clinical inflammation serological markers.** CRP was well correlated with DQ786243 (r = 0.489, *P* = 0.034), CREB (r = 0.500, *P* = 0.029) and Foxp3 (r = 0.546, *P* = 0.016) **(A, B and C)**. Erythrocyte sedimentation (ESR) was also related with the activity of the disease, but no such phenomenon was found **(D, E and F)**. The expression of DQ786243 was correlated with CREB (r = 0.552, *P* = 0.002) and Foxp3 (r = 0.435, *P* = 0.021) at a significant level, but there was no significant correlation between CREB and Foxp3 **(G, H and I)**.

It showed that C-reactive protein (CRP) was well correlated with DQ786243 (r = 0.489, *P* = 0.034), CREB (r = 0.500, *P* = 0.029) and Foxp3 (r = 0.546, *P* = 0.016) (See Figure 2A, B and C). Erythrocyte sedimentation rate (ESR) was also related with the activity of the disease, but no such phenomenon was found (See Figure 2D, E and F). The expression of DQ786243 was correlated with CREB (r = 0.552, *P* = 0.002) and Foxp3 (r = 0.435, *P* = 0.021) at a significant level, but there was no significant correlation between CREB and Foxp3 (See Figure 2G, H and I).

### CREB and Foxp3 expression after DQ786243 transfection

**Figure 3:**
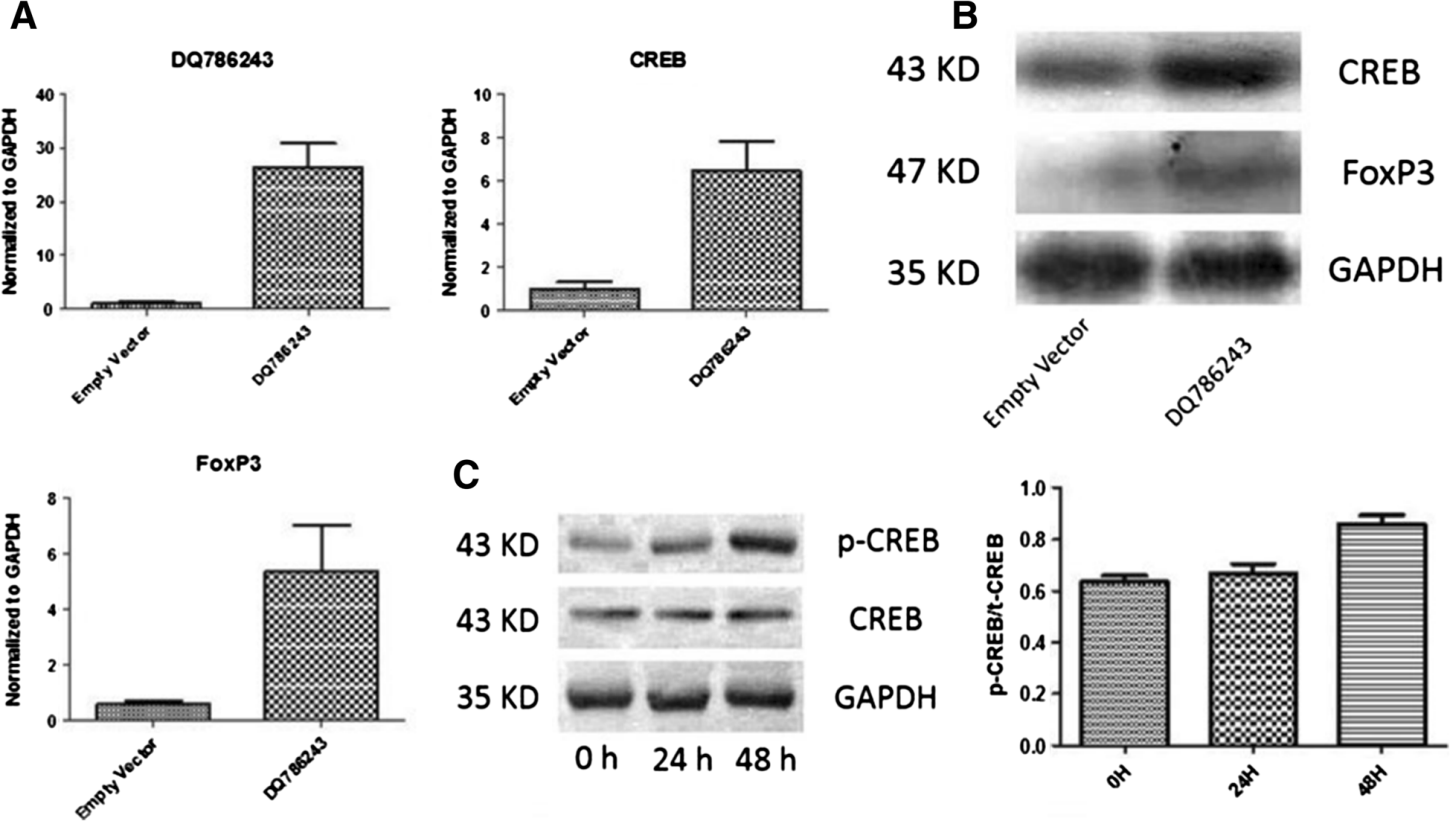
**CREB and Foxp3 expression after DQ786243 transfection.** At 48 hours after DQ786243 transfection, qRT-PCR showed DQ786243 was significantly increased and both CREB and Foxp3 had an increased mRNA expression in Jurkat cells at a significant level **(A)**. Western blot showed the same situation **(B)**. Phosphorylation western blot showed after DQ786243 transfection, the CREB phosphorylation ratio (p-CREB/t-CREB) was increased at 24 hours and 48 hours after transfection **(C)**.

As DQ786243 was correlated the expression of CREB and Foxp3, DQ786243 transient transfection was done in Jurkat cells to see their relations. At 48 hours after DQ786243 transfection, qRT-PCR showed DQ786243 was significantly increased (*P* = 0.005) and both CREB (*P* = 0.017) and Foxp3 (*P* = 0.046) had an increased mRNA expression in Jurkat cells at a significant level (See Figure 3A). Western blot showed the same situation (See Figure 3B). Phosphorylation western blot showed after DQ786243 transfection, the CREB phosphorylation ratio (p-CREB/t-CREB) was increased at 24 hours and 48 hours after transfection (ANOVA, *P* = 0.0043, See Figure 3C).

## Discussion

Tregs are an important subpopulation of T lymphocytes. They involved in many different functions in immune processes and play crucial roles in human disease, such as cancer, allergy. Tregs also play a pivotal role in self-tolerance and resistance to autoimmune diseases. They can down-regulate functions of innate and adaptive immune cells. Immune function disorder has been demonstrated in CD and dysfunction of Tregs may be attributed to this situation, but what makes the irregular functions of Tregs has not been fully understood.

In our research, three genes were detected in CD patients and controls. Early researches demonstrated that CD4+ Foxp3+ Tregs in PBMC from CD patients were decreased [26,27], but Foxp3 transcripts might be similar between patients and controls [26]. These indirect proofs showed that Foxp3 could be lower expressed in CD patients than in controls. In accordance with these results, our study found out there were no higher Foxp3 mRNA expression in CD patients than in controls. In inactive CD samples, the down-regulation of Foxp3 was found. Not like Foxp3, CREB was found up-regulated in PBMCs from CD patients [28]. It was the same as what we found. Actually, there were no close relationship between the expression of Foxp3 and CREB. They might work in different pathways or with other mechanisms, such as phosphorylation. DQ786243, a new lncRNA, could play a critical role in regulating these two genes. It was recently found overexpressed in hepatocellular carcinoma (HCC) [14]. In our study, it was up-regulated in both active and inactive CD patients. DQ786243 was strongly correlated with both Foxp3 and CREB. All the three mRNAs were correlated with CRP, an important serum marker of inflammation. Another indication -- ESR was not. This suggested that DQ786243 could contribute towards development of CD. Malfunction of DQ786243 in CD patient could not be excluded.

We are very curious about how these genes work, but there is no ready-made answer. It is commonly known that transcription factors NFAT, AP1, Sp1, STAT5, Smad3 and CREB-ATF have binding sites on Foxp3 enhancer and promoter [29]. In these transcription factors, CREB works as a part in the enhancer 2 of Foxp3. Some evidences have shown that CREB is critical in Treg generation and maintenance based on TGF-β signaling and T cell receptor (TCR) activation. CREB phosphorylation is activated by TCR activation [30]. Treg-specific demethylated region (TSDR) in Foxp3 locus contains a CREB-activating transcription factor site overlapping a CpG island. The CpG island is found demethylated in Tregs but not in other T cells [15,31-33].

We also wonder the role that lncRNA can play. LncRNA can regulate gene expression via transcription factors. In bladder carcinoma cells, CREB level was significantly down-regulated after knocking down of lncRNA UCA1 [34]. UCA1 alteration paralleled to the expression and phosphorylation of CREB [34]. CREB is not always regulated by lncRNA. At some time, CREB can regulate lncRNA as well. Wang et al. found that lncRNA HULC could be regulated by CREB [35]. MicroRNA (miRNA) - 372 was involved in the up-regulating process. A report showed that lncRNA Tmevpg1 expression contributed to drive Th1-dependent INF-γ expression, but maybe not alone [36]. IFN-γ is critically required in induction of Foxp3 and converts CD4+ CD25- T cells to Tregs [37]. LncRNA could regulate gene expression at different levels, including chromatin modification, transcription and post-transcriptional processing [7].

In our ex vivo study, DQ786243 levels were strongly correlated with CREB levels. In Jurkat cell study, DQ786243 transfection could up-regulate CREB and CREB phosphorylation ratio. CREB plays a critical role in lncRNA regulation. The CREB levels are not so closely related with Foxp3 levels in blood study suggests no direct relationship between CREB expression and Foxp3 level. CREB itself seems not the mediator of DQ786243 to up-regulate Foxp3. The phosphorylation of CREB might play a more important role in the process. Although phosphorylation of CREB can be regulated by DQ786243, further observation is still required. We found that DQ786243 could positively affect the expression of Foxp3. Demethylation of TSDR might join the DQ786243 regulating process and need further research to confirm.

## Conclusion

DQ786243 can be related with severity of CD. It can affect the expression of CREB and Foxp3 through which regulates the function of Treg. CREB itself seems not the mediator of DQ786243 to up-regulate Foxp3. The phosphorylation of CREB might play a more important role in the process.

## Competing interests

The authors declare that they have no competing interests.

## Authors’ contributions

QYQ and SJ gave the conceptions of this article. QYQ and HML drafted the manuscript. QYQ and XAT carried out the molecular studies, ZD participated in the statistics. RZH made critical revise of the manuscript. All authors read and approved the final manuscript.
